# Adult attention-deficit/hyperactivity disorder and bipolar disorder: diagnostic and management challenges

**DOI:** 10.1192/j.eurpsy.2022.1185

**Published:** 2022-09-01

**Authors:** M. Barbosa, R. Guedes

**Affiliations:** Centro Hospitalar de Leiria, Department Of Mental Health And Psychiatry, Leiria, Portugal

**Keywords:** bipolar disorder, adhd

## Abstract

**Introduction:**

Attention-deficit/hyperactivity disorder (ADHD) and bipolar disorder (BD) are neurodevelopmental disorders that commonly persist into adulthood. ADHD in adults can resemble, and often co-occurs with, bipolar disorder (BD), which might lead to diagnostic errors, ineffective treatment and potentially serious adverse consequences.

**Objectives:**

To review on the overlaps and differences in the psychopathology of the two entities and particularities of the management when they occur comorbidely.

**Methods:**

The Medline database through the Pubmed search engine was used with the following keywords: “adhd” and “bipolar disorder”.

**Results:**

ADHD has an estimated prevalence of 10-30% in adults with BD. Despite the symptomatic similarities, there are some important differences. In the ADHD/BD comorbidity, symptoms like attention-deficit, distractibility, irritability, impulsiveness and hyperactivity that may present in (hypo)manic and/or depressive episodes, tend to persist after clinical stabilization. While adult patients with ADHD typically experience ceaseless mental activity and wandering mind, BD patients may have racing thoughts and perceive them as making sense. ADHD patients may have poor socio-occupational achievement that may lead to low self-esteem, low self-confidence and depressed mood. Features like course of illness, psychiatric family history and treatment response may help differentiate the two entities. The treatment must start with mood stabilization and then proceed to the treatment of ADHD symptoms.

**Conclusions:**

A complete clinical history, with particular focus in the neurodevelopmental history, is important but sometimes is not enough for an accurate diagnosis of this comorbidity. As so, clinicians should be aware of the high comorbidity rates to prevent misdiagnosis and provide the best care for both disorders.

**Disclosure:**

No significant relationships.

